# Rebamipide Improves Chronic Inflammation in the Lesser Curvature of the Corpus after *Helicobacter pylori* Eradication: A Multicenter Study

**DOI:** 10.1155/2015/865146

**Published:** 2015-04-28

**Authors:** Tomoari Kamada, Motonori Sato, Tadashi Tokutomi, Tetsuo Watanabe, Takahisa Murao, Hiroshi Matsumoto, Noriaki Manabe, Masanori Ito, Shinji Tanaka, Kazuhiko Inoue, Akiko Shiotani, Takashi Akiyama, Jiro Hata, Ken Haruma

**Affiliations:** ^1^Division of Gastroenterology, Department of Internal Medicine, Kawasaki Medical School, Kurashiki 701-0192, Japan; ^2^Sato Hospital, Kurashiki, Japan; ^3^Tokutomi Clinic, Iwakuni, Japan; ^4^Watanabe Hospital, Kurashiki, Japan; ^5^Division of Endoscopy and Ultrasound, Department of Clinical Pathology and Laboratory Medicine, Kawasaki Medical School, Kurashiki 701-0192, Japan; ^6^Department of Gastroenterology and Metabolism, Graduate School of Biomedical Sciences, Hiroshima University, Japan; ^7^Department of Endoscopy, Hiroshima University Hospital, Hiroshima, Japan; ^8^Department of General Medicine, Kawasaki Medical School, Kurashiki 701-0192, Japan; ^9^Department of Pathology, Kawasaki Medical School, Kurashiki 701-0192, Japan

## Abstract

*Background and Aim*. Although many epidemiologic studies have shown that *Helicobacter pylori* eradication has prophylactic effects on gastric cancer, it does not completely eliminate the risk of gastric cancer. We aimed to investigate the changes in histological gastritis in patients receiving rebamipide treatment after *H. pylori* eradication. *Methods*. 206 patients who had undergone *H. pylori* eradication were evaluated. Of these, 169 patients who achieved successful eradication were randomly allocated to 2 groups: the rebamipide group (*n* = 82) and the untreated group (*n* = 87). The primary endpoints were histopathological findings according to the updated Sydney system at the start of the study and after 1 year. *Results*. Final assessment for histological gastritis was possible in 50 cases from the rebamipide group and 53 cases from the untreated group. The activity and atrophy improved in both the rebamipide and untreated groups, and no significant intergroup differences were observed. Chronic inflammation affecting the lesser curvature of the corpus was significantly improved in the rebamipide group compared to in the untreated group (1.12 ± 0.08 versus 1.35 ± 0.08; *P* = 0.043). *Conclusions*. Rebamipide treatment after *H. pylori* eradication alleviated chronic inflammation in the lesser curvature of the corpus compared to that in the untreated group. This trial is registered with UMIN000002369.

## 1. Introduction

Infection is a major etiology of human cancer, and prevention and treatment of infectious organisms, including viruses, bacteria, and parasites, have been shown to have significant beneficial effects on cancer prevention. Gastric cancer is the second most common cause of cancer-related death worldwide and was the most common cause of cancer-related death in Japan until it was replaced by lung cancer in 1995 [1]. After the discovery of* Helicobacter pylori* in 1983 [2], causal relationships between this bacterium and gastritis and gastric cancer have been elucidated. Results from animal and epidemiological studies suggest that* H. pylori* infection and the subsequent gastritis promote the development of gastric cancer in the infected mucosa. The evidence for this causal relationship between* H. pylori* and gastric cancer [3–6] is so overwhelming that the World Health Organization and the International Agency for Research on Cancer have declared* H. pylori* as a group 1 carcinogen for gastric cancer [7, 8].

In 2008, a randomized multicenter clinical study conducted in Japan revealed that eradication of* H. pylori* reduced the incidence of secondary gastric cancer by approximately two-thirds after endoscopic mucosal resection of early gastric cancer [9], suggesting the usefulness of* H. pylori* eradication for prevention of gastric cancer. As a result of this study, the Japanese Society for Helicobacter Research published a guideline in 2009, in which it recommended that all* H. pylori*-infected people should receive bacterial eradication therapy [10]. However, our previous study also showed that* H. pylori* eradication did not completely eliminate the risk of gastric cancer [11], and considering the occasional development of gastric cancer after* H. pylori* eradication, it is important to evaluate the need for continuing medication in patients undergoing this treatment.

The results of our previous studies indicated that residual inflammation in the lesser curvature of the corpus after* H. pylori* eradication is a risk factor for the development of metachronous gastric cancer [12, 13]. Rebamipide, a gastroprotective drug, was widely developed in Japan for the treatment of peptic ulcer disease, chronic gastritis, endoscopic submucosal dissection-induced ulcers, and after treatment of* H. pylori* eradication [14–16]. Our previous study [17] showed that long-term rebamipide treatment improved histologic gastritis in terms of mononuclear cell infiltration into the antrum and corpus and resulted in decreased serum gastrin levels in patients with* H. pylori*-associated gastritis for 1 year. In this study, to further elucidate the mechanisms by which rebamipide improves chronic inflammation, we aimed to investigate the changes in histological gastritis and subjective symptoms in patients receiving rebamipide treatment after* H. pylori* eradication.

## 2. Patients and Methods

This multicenter, randomized, controlled clinical trial was conducted in 6 centers throughout Japan between November 2009 and April 2012. Criteria for inclusion were patients aged 18 years or older diagnosed with early gastric cancer, gastric ulcers, or atrophic gastritis by endoscopy and proven to be* H. pylori*-positive. The exclusion criteria were as follows: (i) previous history of* H. pylori* eradication; (ii) patients who had been administered drugs that may have affected the evaluation 2 weeks before the enrollment (nonsteroidal anti-inflammatory drugs [NSAIDs], proton pump inhibitors, or antibiotics); (iii) severe heart or pulmonary disease; (iv) pregnancy; (v) allergic habitus; and (vi) other situations that the investigators considered unsuitable for the study. The institutional review board of our hospital approved the study protocol, which conforms to the provisions of the Declaration of Helsinki (as revised in Tokyo 2004), and all patients provided written informed consent to participate in the study.

Upon enrollment in the trial, all patients received eradication triple therapy with 20 mg of omeprazole, 750 mg of amoxicillin, and 200 mg of clarithromycin twice daily for 1 week. Eradication of* H. pylori* infection was determined by the ^13^C-urea breath test (UBT) (Δ ^13^C cutoff value: 2.5%) 8 weeks after the eradication [18]. Patients with confirmed eradication were randomly allocated to two groups: the rebamipide group (100 mg of rebamipide three times daily) or the untreated group (no treatment) for 1 year. During the study period, the use of all other antiulcer agents, antibacterial agents, and NSAIDs was prohibited.

Prior to the start of the study, and at 1 year after the study start, all patients were subjected to endoscopy with histology, frequency scale for the symptoms of gastroesophageal reflux disease (FSSG) questionnaire, and serological marker (serum gastrin and pepsinogen) evaluations. If the first course of eradication therapy failed, the patients received a second eradication course with 20 mg of omeprazole, 750 mg of amoxicillin, and 250 mg of metronidazole twice daily for an additional week. After assessment of the treatment by ^13^C-UBT, the patients who succeeded in the second eradication therapy were randomly allocated to the two groups; however, patients who failed the treatment again were excluded from the study. The primary endpoints were assessment of the histological findings according to the updated Sydney system [19] at the start of the study and after 1 year. The secondary endpoints were assessment of FSSG questionnaires and serological markers.

Three biopsy specimens were obtained from the greater curvature of the middle antrum and the lesser and greater curvatures of the middle corpus for evaluation of gastritis. Biopsy specimens were cut into 4 *μ*m thick slices and evaluated using hematoxylin and eosin or Gimenez staining. The histological specimens were independently assessed by two gastroenterologists with no knowledge of the clinical findings of the patients. Disagreements were resolved by joint review and discussion. The presence of* H. pylori* was observed using Gimenez staining. Neutrophil infiltration, chronic inflammation, atrophy, and intestinal metaplasia were scored on a scale of 0 to 3 according to the updated Sydney classification system: 0 = normal, 1 = mild, 2 = moderate, and 3 = severe.

The FSSG was used to evaluate subjective symptoms of reflux esophagitis, as a Japanese gastroesophageal reflux disease-specific, self-administered questionnaire that corresponds to multidimensional symptoms [20, 21]. The FSSG consists of 12 questions, with the score of each question determined according to the frequency of symptoms (never: 0; occasionally: 1; sometimes: 2; often: 3; always: 4). The scores for each question were calculated and evaluated as a total score. The questions were classified into two types: reflux-related symptoms and dysmotility-related symptoms. This scale is characterized by a simple form and can be applied for the early diagnosis of gastroesophageal reflux disease and functional dyspepsia and to evaluate the efficacy of medical therapy [22].

Fasting sera were collected at the start of the study and after 1 year and stored at −80°C until use. Secondary objectives included measurement of serum gastrin (Gastrin RIA Kit II; Dainabot, Tokyo, Japan) and pepsinogen I and II levels (LZ test; Eiken, Tokyo, Japan) by radioimmunoassay and enzyme immunoassay, respectively [23]. Serum gastrin ≤ 200 pg/mL was regarded as normal, and both PG I ≤ 70 ng/mL and PG I/II ≤ 3 were regarded as PG-positive, indicative of gastric mucosal atrophy [24]. PGII concentrations of 12 ng/mL or more or I/II ratio of 4.0 or less were used as the cutoff points for the diagnosis of* H. pylori* infection [25].

## 3. Statistical Evaluation

The demographic characteristics, FSSG scores, serum markers, and histological grading before treatment and after treatment were compared between the two groups using Student's *t*-test. Within each group, the FSSG scores and serum markers were compared before and after therapy by using the paired *t*-test. The results are expressed as mean ± standard error. *P* values < 0.05 were considered statistically significant. All statistical computations were performed using SPSS (SPSS Inc., Chicago, IL, USA).

## 4. Results

**Figure 1:**
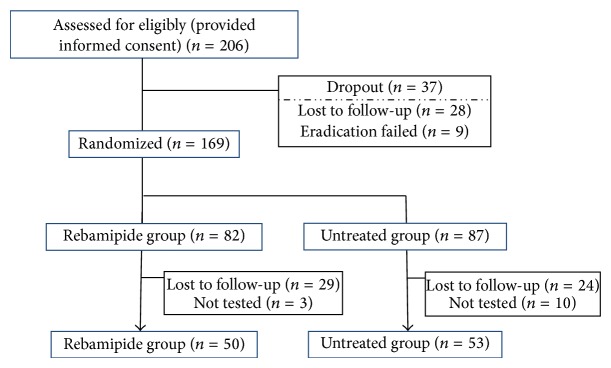
Flowchart of the study participants. Analysis at 1 year after the start of the study was possible in 50 cases from the rebamipide group and 53 cases from the untreated group.

Of the 206 patients in this study who underwent eradication therapy, 28 were lost to follow-up and 9 failed the eradication assessment. The remaining 169 patients, who were confirmed to have achieved successful eradication, were randomly allocated to two groups: the rebamipide group (*n* = 82) and the untreated group (*n* = 87). During the treatment, 29 patients were lost to follow-up and 3 were not tested for histological assessment in the rebamipide group; in the untreated group, 24 patients were lost to follow-up and 10 were not tested for histological assessment. Finally, assessment for histological gastritis, FSSG scores, and serological markers before and 1 year after the start of the study was possible in 50 cases from the rebamipide group and 53 cases from the untreated group (Figure 1).

**Figure 2:**
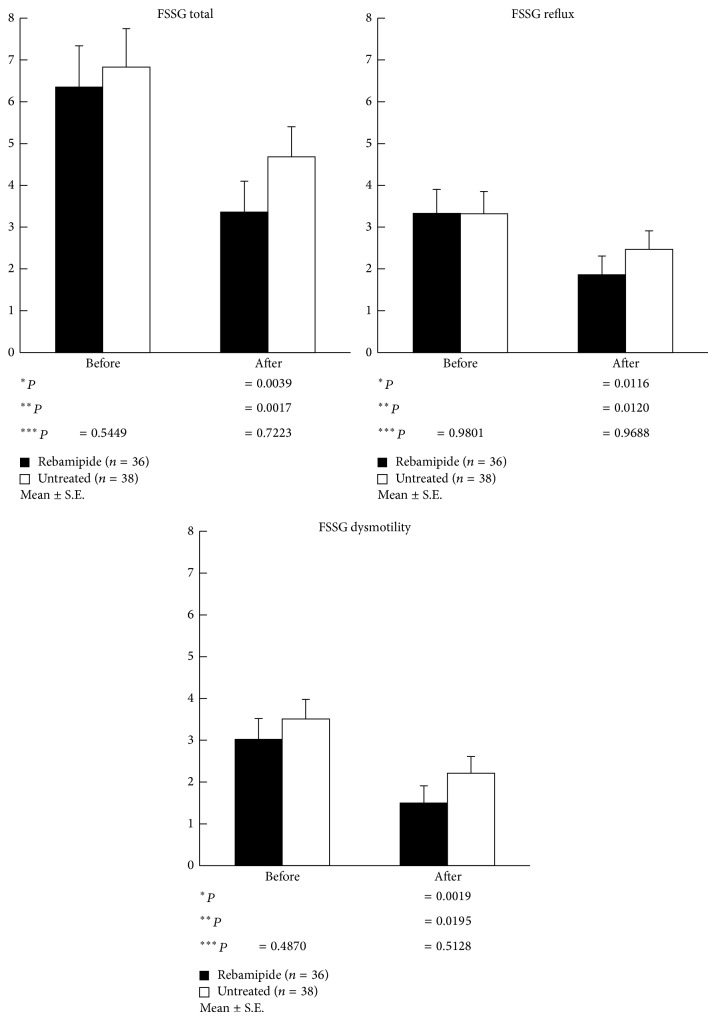
Mean frequency scale for the symptoms of gastroesophageal reflux disease (FSSG) scores in the two groups before and after treatment. The FSSG scores, which included the reflux- and dysmotility-related scores, significantly improved in both groups after* H. pylori* eradication, but no significant intergroup differences were observed. ^∗^Compared with baseline (rebamipide group). ^∗∗^Compared with baseline (untreated group). ^∗∗∗^Rebamipide versus untreated group.

**Figure 3:**
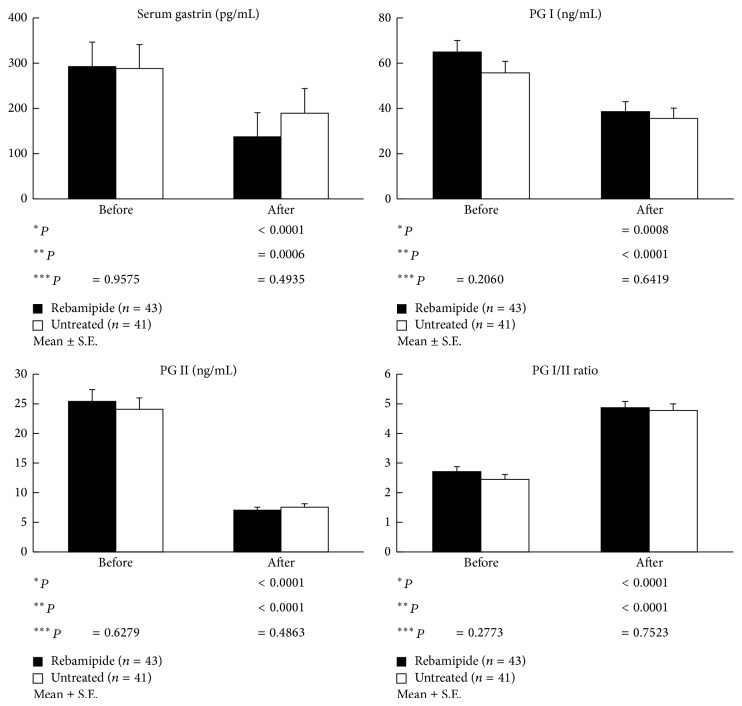
Differences in the serum gastrin and pepsinogen (PG) levels between the two groups before and after treatment. The serum gastrin and pepsinogen levels significantly improved in both groups after* H. pylori* eradication, but no significant intergroup differences were observed. ^∗^Compared with baseline (rebamipide group). ^∗∗^Compared with baseline (untreated group). ^∗∗∗^Rebamipide versus untreated group.

Comparison of the demographic characteristics showed that there were no significant differences between the rebamipide and untreated groups in terms of gender, age, alcohol and smoking habits, and past medical history, as shown in Table 1. However, the prevalence of gastric ulcer in the rebamipide group (15.6%) was significantly higher, and atrophic gastritis (78.0%) in the rebamipide group was significantly lower than that of the untreated group. In addition, there were no significant differences between the two groups in terms of the FSSG score, serum gastrin and pepsinogen levels, and histological assessment (neutrophil infiltration, chronic inflammation, atrophy, and intestinal metaplasia) before the treatment (Figures 2 and 3).

The FSSG scores, including both the reflux and dysmotility-related scores, significantly improved in both groups after* H. pylori* eradication, and no significant intergroup differences were observed (Figure 2). Similarly, the serum gastrin and pepsinogen levels also significantly decreased in both groups after* H. pylori* eradication, with no significant intergroup differences noted (Figure 3). With the exception of intestinal metaplasia, histological gastritis significantly improved in both groups after* H. pylori* eradication (Tables 2 and 3). Chronic inflammation affecting the lesser curvature of the corpus after* H. pylori* eradication was significantly improved in the rebamipide group compared to in the untreated group (1.12 ± 0.56 versus 1.35 ± 0.52; *P* < 0.05) (Table 4).

## 5. Discussion

Our study demonstrated that* H. pylori* eradication significantly improved not only chronic inflammation but also mucosal atrophy for only 1 year. Additionally, our study also showed that rebamipide treatment after* H. pylori* eradication significantly alleviated chronic inflammation in the lesser curvature of the corpus compared to the untreated group.

Several animal studies have demonstrated an association between gastric carcinogenesis and* H. pylori* infection [26, 27], and a large meta-analysis of several epidemiological studies confirmed the association between the organism and gastric cancer development [3]. In 2001, a prospective observational study showed that 2.9% of patients with* H. pylori* infection develop gastric cancer, in contrast to 0% of* H. pylori*-negative patients [6]. Additionally, Fukase et al. [9] conducted a multicenter, open-label, randomized trial, which recruited 544 patients with early gastric cancer treated by endoscopic resection who were evenly randomized to either an eradication regimen or control group. They found that* H. pylori* eradication could reduce the subsequent development of metachronous gastric cancer after endoscopic resection of early gastric cancer. However, the effects of suppressing gastric cancer by eradication of* H. pylori* are somewhat limited, as gastric cancer is known to recur even after eradication.

It is well known that histological gastritis, including mucosal atrophy, activity, and inflammation, gradually improves after* H. pylori* eradication. Kodama et al. [28] showed that, 10 years after* H. pylori* eradication, the inflammation, activity, and atrophy scores at all sites and intestinal metaplasia in the lesser curvature of the corpus gradually and significantly decreased. These results suggest that the improvement of gastric atrophy and intestinal metaplasia might be associated with the reduction of gastric cancer occurrence. In our study, except for intestinal metaplasia, histological gastritis after* H. pylori* eradication significantly improved at all sites in both the rebamipide and the untreated groups. In addition, in the rebamipide group, chronic inflammation in the lesser curvature of the corpus was significantly improved compared to that in the untreated group, suggesting that continuous treatment of rebamipide for 1 year after eradication might contribute to improving the chronic inflammation. Moreover, our data showed that the serum gastrin was significantly lower, and the pepsinogen I/II ratio was significantly higher than before eradication, confirming the improvement of histological gastritis by* H. pylori* eradication.

The results of our previous studies suggested that persistent corpus gastritis after eradication of* H. pylori* might be a risk factor for cancer development [12, 13]. For example, after eradication, the scores for inflammation in the corpus were significantly higher in the gastric cancer group than in the control group, indicating that treatment of corporal inflammation after eradication might contribute to preventing the development of* de novo* gastric cancer. Rebamipide's mechanisms of actions are different from antisecretory drugs; it has stimulation of prostaglandin and mucus glycoprotein synthesis [29], free radical scavenging activity, antioxidant activity [30], and anti-inflammatory activity [17], although it has no effect on the eradication of* H. pylori*. Therefore, these mechanisms of rebamipide may contribute to improving the residual inflammation in the lesser curvature of the corpus after* H. pylori* eradication which is a risk factor for the development of metachronous gastric cancer.

Although eradication of* H. pylori* infection in patients with functional dyspepsia continues to be a matter of debate,* H. pylori* eradication has been shown to have statistically significant effects on the symptom relief in these patients [31–33]. A meta-analysis on 17 randomized controlled trials (*n* = 3186) found a small but statistically significant benefit of* H. pylori* eradication therapy at 3–12 months, with a relative risk reduction of 8% (95% confidence interval [CI], 3–12%) and a number needed to treat (NNT) of 18 (95% CI, 12–48) [32]. Recently, Zhao et al. analyzed 14 randomized controlled trials (*n* = 2993) on dyspeptic syndrome in Asian, European, and American populations and found that eradication therapy was almost 1.4 times more likely to result in symptom improvement compared to a placebo or other traditional therapies (odds ratio, 1.38; 95% CI, 1.18–1.62; *P* < 0.0001; NNT, 15) [33]. Similarly, in the present study conducted in a Japanese population, reflux and dyspeptic symptoms were found to improve 1 year after eradication of* H. pylori*, especially in the rebamipide group.

There is one limitation to this study. In our subjects, the percentage of patients with gastric ulcer in the rebamipide group (15.6%) was significantly higher than that in the untreated group (4.6%). Otherwise, the percentage of patients with atrophic gastritis (78.0%) was significantly lower than that of the untreated group (92.0%). These differences may have introduced a minor bias into our results.

In conclusion, our study demonstrated that* H. pylori* eradication significantly improved not only chronic inflammation but also mucosal atrophy for only 1 year, and rebamipide treatment after* H. pylori* eradication significantly alleviated chronic inflammation in the lesser curvature of the corpus compared to the untreated group. Long-term rebamipide treatment after* H. pylori* eradication might be able to prevent the onset of gastric cancer; and large-scale, long-term endoscopic surveillance studies are therefore required in the future to assess the effects of rebamipide after* H. pylori* eradication on the prevention of gastric cancer.

## Figures and Tables

**Table 1:** Demographic characteristics of the patients.

	Rebamipide group (*n* = 82)	Untreated group (*n* = 87)	*P* value
Mean age (range)	57.7 ± 1.5 (22–85)	56.7 ± 1.4 (27–80)	0.6138
Gender (male/female)	42/40	36/51	0.1997
Endoscopic diagnosis	Gastric ulcer, 13 Atrophic gastritis, 64 Early GC after ESD, 5	Gastric ulcer, 4 Atrophic gastritis, 80 Early GC after ESD, 3	0.0317
Alcohol (yes/no)	31/40 (unknown, 11)	23/55 (unknown, 9)	0.0722
Smoking (yes/no)	11/59 (unknown, 12)	8/69 (unknown, 10)	0.3365
Past history (yes/no)	15/36 (unknown, 31)	17/32 (unknown, 38)	0.5714

Data are presented as mean ± standard error or as *n*. GC: gastric cancer and ESD: endoscopic submucosal dissection.

**Table 2:** Comparison of histological gastritis before and after *H. pylori *eradication in the rebamipide group.

		Before eradication	After eradication	*P* value
GC of the middle antrum	Activity	1.00 ± 0.12 (48)	0.00 ± 0.00 (47)	<0.0001
	Inflammation	2.44 ± 0.10 (48)	1.21 ± 0.08 (47)	<0.0001
	Atrophy	1.27 ± 0.07 (48)	0.96 ± 0.10 (47)	0.0270
	Intestinal metaplasia	0.69 ± 0.16 (48)	0.43 ± 0.11 (47)	0.0887

GC of the middle corpus	Activity	1.08 ± 0.11 (49)	0.02 ± 0.01 (47)	<0.0001
	Inflammation	2.24 ± 0.09 (49)	1.11 ± 0.10 (47)	<0.0001
	Atrophy	0.90 ± 0.09 (49)	0.34 ± 0.08 (47)	<0.0001
	Intestinal metaplasia	0.14 ± 0.09 (49)	0.19 ± 0.09 (47)	0.5039

LC of the middle corpus	Activity	1.00 ± 0.11 (48)	0.00 ± 0.00 (49)	<0.0001
	Inflammation	2.29 ± 0.10 (48)	1.12 ± 0.08 (49)	<0.0001
	Atrophy	1.17 ± 0.09 (48)	0.71 ± 0.10 (49)	0.0005
	Intestinal metaplasia	0.85 ± 0.17 (48)	0.84 ± 0.16 (49)	0.6950

Data are presented as mean ± standard error (*n*). GC: greater curvature and LC: lesser curvature.

**Table 3:** Comparison of histological gastritis before and after *H. pylori *eradication in the untreated group.

		Before eradication	After eradication	*P* value
GC of the middle antrum	Activity	0.94 ± 0.11 (52)	0.06 ± 0.02 (52)	<0.0001
	Inflammation	2.31 ± 0.10 (52)	1.37 ± 0.07 (52)	<0.0001
	Atrophy	1.25 ± 0.07 (52)	0.96 ± 0.10 (52)	0.0098
	Intestinal metaplasia	0.73 ± 0.16 (52)	0.44 ± 0.11 (52)	0.0719

GC of the middle corpus	Activity	1.21 ± 0.10 (52)	0.00 ± 0.00 (49)	<0.0001
	Inflammation	2.35 ± 0.09 (52)	1.29 ± 0.09 (49)	<0.0001
	Atrophy	1.02 ± 0.09 (52)	0.36 ± 0.08 (49)	<0.0001
	Intestinal metaplasia	0.23 ± 0.08 (52)	0.27 ± 0.09 (49)	0.7988

LC of the middle corpus	Activity	0.94 ± 0.11 (48)	0.00 ± 0.00 (49)	<0.0001
	Inflammation	2.40 ± 0.10 (48)	1.35 ± 0.08 (49)	<0.0001
	Atrophy	1.08 ± 0.09 (48)	0.80 ± 0.10 (49)	0.0178
	Intestinal metaplasia	0.96 ± 0.17 (48)	0.78 ± 0.16 (49)	0.3537

Data are presented as mean ± standard error (*n*). GC: greater curvature and LC: lesser curvature.

**Table 4:** Comparison of histological gastritis after *H. pylori *eradication between the two groups.

		Rebamipide group (*n* = 50)	Untreated group (*n* = 53)	*P* value
GC of the middle antrum	Activity	0.00 ± 0.00 (47)	0.06 ± 0.24 (52)	0.0963
	Inflammation	1.21 ± 0.08 (47)	1.37 ± 0.07 (52)	0.1457
	Atrophy	0.96 ± 0.10 (47)	0.96 ± 0.10 (52)	0.9765
	Intestinal metaplasia	0.43 ± 0.11 (47)	0.44 ± 0.11 (52)	0.9146

GC of the middle corpus	Activity	0.02 ± 0.01 (47)	0.00 ± 0.00 (49)	0.3077
	Inflammation	1.11 ± 0.10 (47)	1.29 ± 0.09 (49)	0.1839
	Atrophy	0.34 ± 0.08 (47)	0.36 ± 0.08 (49)	0.8094
	Intestinal metaplasia	0.19 ± 0.09 (47)	0.27 ± 0.09 (49)	0.5751

LC of the middle corpus	Activity	0.00 ± 0.00 (49)	0.00 ± 0.00 (49)	—
	Inflammation	1.12 ± 0.08 (49)	1.35 ± 0.08 (49)	0.0437
	Atrophy	0.71 ± 0.10 (49)	0.80 ± 0.10 (49)	0.5518
	Intestinal metaplasia	0.84 ± 0.16 (49)	0.78 ± 0.16 (49)	0.7808

Data are presented as mean ± standard error (*n*). GC: greater curvature and LC: lesser curvature.

## References

[1] Marugame T., Matsuda T., Kamo K.-I., Katanoda K., Ajiki W., Sobue T. (2007). Cancer Incidence and incidence rates in Japan in 2001 based on the data from 10 population-based cancer registries. *Japanese Journal of Clinical Oncology*.

[2] Warren J. R., Marshall B. J. (1983). Unidentified curved bacilli on gastric epithelium in active chronic gastritis. *The Lancet*.

[3] Huang J. Q., Sridhar S., Chen Y., Hunt R. H. (1998). Meta-analysis of the relationship between *Helicobacter pylori* seropositivity and gastric cancer. *Gastroenterology*.

[4] Nomura A., Stemmermann G. N., Chyou P.-H., Kato I., Perez-Perez G. I., Blaser M. J. (1991). *Helicobacter pylori* infection and gastric carcinoma among Japanese Americans in Hawaii. *The New England Journal of Medicine*.

[5] Parsonnet J., Friedman G. D., Vandersteen D. P. (1991). *Helicobacter pylori* infection and the risk of gastric carcinoma. *The New England Journal of Medicine*.

[6] Uemura N., Okamoto S., Yamamoto S. (2001). *Helicobacter pylori* infection and the development of gastric cancer. *The New England Journal of Medicine*.

[7] IARC Working Group on the Evaluation of Carcinogenic Risks to Humans (1994). *Schistosomes*, liver flukes and *Helicobacter pylori*. *IARC Monographs on the Evaluation of Carcinogenic Risks to Humans*.

[8] IARC Working Group on the Evaluation of Carcinogenic Risks to Humans (2012). Biological agents. Volume 100 B. A review of human carcinogens. *IARC Monographs on the Evaluation of Carcinogenic Risks to Humans*.

[9] Fukase K., Kato M., Kikuchi S. (2008). Effect of eradication of *Helicobacter pylori* on incidence of metachronous gastric carcinoma after endoscopic resection of early gastric cancer: an open-label, randomised controlled trial. *The Lancet*.

[10] Asaka M., Kato M., Takahashi S.-I. (2010). Guidelines for the management of *Helicobacter pylori* infection in Japan: 2009 revised edition. *Helicobacter*.

[11] Kamada T., Hata J., Sugiu K. (2005). Clinical features of gastric cancer discovered after successful eradication of *Helicobacter pylori*: results from a 9-year prospective follow-up study in Japan. *Alimentary Pharmacology and Therapeutics*.

[12] Shiotani A., Uedo N., Iishi H. (2007). Re-expression of sonic hedgehog and reduction of CDX2 after *Helicobacter pylori* eradication prior to incomplete intestinal metaplasia. *International Journal of Cancer*.

[13] Shiotani A., Uedo N., Iishi H. (2008). Predictive factors for metachronous gastric cancer in high-risk patients after successful *Helicobacter pylori* eradication. *Digestion*.

[14] Higuchi K., Takeuchi T., Uedo N. (2014). Efficacy and safety of 1-week *Helicobacter pylori* eradication therapy and 7-week rebamipide treatment after endoscopic submucosal dissection of early gastric cancer in comparison with 8-week PPI standard treatment: a randomized, controlled, prospective, multicenter study. *Gastric Cancer*.

[15] Takayama M., Matsui S., Kawasaki M. (2013). Efficacy of treatment with rebamipide for endoscopic submucosal dissection-induced ulcers. *World Journal of Gastroenterology*.

[16] Song K. H., Lee Y. C., Fan D.-M. (2011). Healing effects of rebamipide and omeprazole in *Helicobacter pylori*-positive gastric ulcer patients after eradication therapy: a randomized double-blind, multinational, multi-institutional comparative study. *Digestion*.

[17] Haruma K., Ito M., Kido S. (2002). Long-term rebamipide therapy improves *Helicobacter pylori*-associated chronic gastritis. *Digestive Diseases and Sciences*.

[18] Chen X., Haruma K., Kamada T. (2000). Factors that affect results of the 13C urea breath test in Japanese patients. *Helicobacter*.

[19] Dixon M. F., Genta R. M., Yardley J. H., Correa P. (1996). Classification and grading of gastritis. The updated Sydney System. International Workshop on the Histopathology of Gastritis, Houston 1994. *The American Journal of Surgical Pathology*.

[20] Kusano M., Shimoyama Y., Sugimoto S. (2004). Development and evaluation of FSSG: frequency scale for the symptoms of GERD. *Journal of Gastroenterology*.

[21] Kusano M., Hosaka H., Kawada A. (2012). Development and evaluation of a modified Frequency Scale for the Symptoms of Gastroesophageal Reflux Disease to distinguish functional dyspepsia from non-erosive reflux disease. *Journal of Gastroenterology and Hepatology*.

[22] Hongo M., Miwa H., Kusano M., J-FAST Group (2012). Symptoms and quality of life in underweight gastroesophageal reflux disease patients and therapeutic responses to proton pump inhibitors. *Journal of Gastroenterology and Hepatology*.

[23] Yoshihara M., Sumii K., Haruma K. (1998). Correlation of ratio of serum pepsinogen I and II with prevalence of gastric cancer and adenoma in Japanese subjects. *American Journal of Gastroenterology*.

[24] Miki K., Morita M., Sasajima M., Hoshina R., Kanda E., Urita Y. (2003). Usefulness of gastric cancer screening using the serum pepsinogen test method. *The American Journal of Gastroenterology*.

[25] Kiyohira K., Yoshihara M., Ito M., Haruma K., Tanaka S., Chayama K. (2003). Serum pepsinogen concentration as a marker of *Helicobacter pylori* infection and the histologic grade of gastritis; evaluation of gastric mucosa by serum pepsinogen levels. *Journal of Gastroenterology*.

[26] Sugiyama A., Maruta F., Ikeno T. (1998). *Helicobacter pylori* infection enhances N-methyl-N-nitrosourea-induced stomach carcinogenesis in the Mongolian gerbil. *Cancer Research*.

[27] Watanabe T., Tada M., Nagi H., Sasaki S., Nakao M. (1998). *Helicobacter pylori* infection induces gastric cancer in Mongolian gerbils. *Gastroenterology*.

[28] Kodama M., Murakami K., Okimoto T. (2012). Ten-year prospective follow-up of histological changes at five points on the gastric mucosa as recommended by the updated Sydney system after *Helicobacter pylori* eradication. *Journal of Gastroenterology*.

[29] Kleine A., Kluge S., Peskar B. M. (1993). Stimulation of prostaglandin biosynthesis mediates gastroprotective effect of rebamipide in rats. *Digestive Diseases and Sciences*.

[30] Naito Y., Yoshikawa T., Tanigawa T. (1995). Hydroxyl radical scavenging by rebamipide and related compounds: electron paramagnetic resonance study. *Free Radical Biology and Medicine*.

[31] Moayyedi P., Deeks J., Talley N. J., Delaney B., Forman D. (2003). An update of the Cochrane systematic review of *Helicobacter pylori* radication therapy in nonulcer dyspepsia: resolving the discrepancy between systematic reviews. *The American Journal of Gastroenterology*.

[32] Moayyedi P., Soo S., Deeks J. (2005). Eradication of *Helicobacter pylori* for non-ulcer dyspepsia. *Cochrane Database of Systematic Reviews*.

[33] Zhao B., Zhao J., Cheng W.-F. (2014). Efficacy of *Helicobacter pylori* eradication therapy on functional dyspepsia: a meta-analysis of randomized controlled studies with 12-month follow-up. *Journal of Clinical Gastroenterology*.

